# Long-Term Efficacy of a Mobile Mental Wellness Program: Prospective Single-Arm Study

**DOI:** 10.2196/54634

**Published:** 2024-06-27

**Authors:** Meaghan McCallum, Matthew Baldwin, Paige Thompson, Kelly Blessing, Maria Frisch, Annabell Ho, Matthew Cole Ainsworth, Ellen Siobhan Mitchell, Andreas Michaelides, Christine N May

**Affiliations:** 1 Academic Research Noom, Inc New York City, NY United States

**Keywords:** mHealth, psychological distress, Noom Mood, digital mental wellness programs, mobile phone

## Abstract

**Background:**

Rising rates of psychological distress (symptoms of depression, anxiety, and stress) among adults in the United States necessitate effective mental wellness interventions. Despite the prevalence of smartphone app–based programs, research on their efficacy is limited, with only 14% showing clinically validated evidence. Our study evaluates Noom Mood, a commercially available smartphone-based app that uses cognitive behavioral therapy and mindfulness-based programming. In this study, we address gaps in the existing literature by examining postintervention outcomes and the broader impact on mental wellness.

**Objective:**

Noom Mood is a smartphone-based mental wellness program designed to be used by the general population. This prospective study evaluates the efficacy and postintervention outcomes of Noom Mood. We aim to address the rising psychological distress among adults in the United States.

**Methods:**

A 1-arm study design was used, with participants having access to the Noom Mood program for 16 weeks (N=273). Surveys were conducted at baseline, week 4, week 8, week 12, week 16, and week 32 (16 weeks’ postprogram follow-up). This study assessed a range of mental health outcomes, including anxiety symptoms, depressive symptoms, perceived stress, well-being, quality of life, coping, emotion regulation, sleep, and workplace productivity (absenteeism or presenteeism).

**Results:**

The mean age of participants was 40.5 (SD 11.7) years. Statistically significant improvements in anxiety symptoms, depressive symptoms, and perceived stress were observed by week 4 and maintained through the 16-week intervention and the 32-week follow-up. The largest changes were observed in the first 4 weeks (29% lower, 25% lower, and 15% lower for anxiety symptoms, depressive symptoms, and perceived stress, respectively), and only small improvements were observed afterward. Reductions in clinically relevant anxiety (7-item generalized anxiety disorder scale) and depression (8-item Patient Health Questionnaire depression scale) criteria were also maintained from program initiation through the 16-week intervention and the 32-week follow-up. Work productivity also showed statistically significant results, with participants gaining 2.57 productive work days from baseline at 16 weeks, and remaining relatively stable (2.23 productive work days gained) at follow-up (32 weeks). Additionally, effects across all coping, sleep disturbance (23% lower at 32 weeks), and emotion dysregulation variables exhibited positive and significant trends at all time points (15% higher, 23% lower, and 25% higher respectively at 32 weeks).

**Conclusions:**

This study contributes insights into the promising positive impact of Noom Mood on mental health and well-being outcomes, extending beyond the intervention phase. Though more rigorous studies are necessary to understand the mechanism of action at play, this exploratory study addresses critical gaps in the literature, highlighting the potential of smartphone-based mental wellness programs to lessen barriers to mental health support and improve diverse dimensions of well-being. Future research should explore the scalability, feasibility, and long-term adherence of such interventions across diverse populations.

## Introduction

Psychological distress, encompassing nonspecific symptoms of stress, anxiety, and depression, has been on the rise in the United States since 1999. More specifically, a 40% increase in prevalence was found from 2000 to 2018 [1-4] and was exacerbated by the COVID-19 pandemic [5-9]. One 2020 meta-analysis found the prevalence of stress in the general population to be between 29.6% and 33.7% [5]. This distress correlates with personal and occupational impairment, chronic medical disorders, adverse health behaviors, and increased mortality risk from diseases such as cardiovascular disease, cancer, and liver disease [10-12]. Depression and anxiety symptoms also predict absenteeism (missed work days) and presenteeism (low productivity days), costing an estimated 3% to 4% of the gross national product [13-16].

Despite the high prevalence, a large portion of affected adults do not receive necessary mental health support. Common barriers to mental health support in the United States include a desire for autonomy in the process, fear of social stigma, and lack of access (financially and structurally) [17-19]. Smartphone app–based mental wellness programs offer a promising solution, and the benefits of mobile mental wellness programs include increased convenience, adherence, personalization, social support, and cost-effectiveness [20-22]. However, research on their effectiveness is lacking [23], with only 14% showing clinically validated evidence of effectiveness [18], particularly among commercially available programs.

In response to the scarcity of clinical validation among these programs, we previously evaluated the feasibility and short-term efficacy of Noom Mood [24], a commercially available, smartphone-based mental wellness program that provides educational content, skills-based activities, and nonclinical coaching support to help users manage stress and symptoms of anxiety. In this study, we extend our initial findings (at 4 weeks) to the full length of the intervention (at 16 weeks) and postintervention (at 32 weeks) in a larger sample. We also aim to contribute to important gaps within the literature by increasing our understanding of the efficacy and outcome maintenance associated with mobile mental wellness programs.

Research on the maintenance of outcomes after completing mobile mental wellness programs is currently scarce and is essential to inform postintervention support and establish long-term efficacy. Economides and colleagues [25] provide some evidence that a reduction in anxiety and depressive symptoms can be maintained at 6 months post intervention. However, their analysis was retrospective, and follow-up assessments were limited. High-quality, long-term postintervention data are needed to better understand the full clinical utility of mobile mental wellness programming for the general population [26]. In addition, there are gaps in the existing literature looking at overall mental wellness, beyond mental health-related symptoms (eg, emotion regulation, coping, or quality of life). Despite theoretical and empirical assertions that smartphone-based programs can indirectly help improve mental wellness, such outcomes have not been widely studied [27,28].

This 1-arm prospective study examines the efficacy and postintervention outcomes of a commercially available mental wellness program on a broad range of mental health outcomes: symptoms of anxiety, stress, and depression, as well as well-being, quality of life, coping skills, emotion regulation, sleep, and workplace presenteeism and absenteeism. In doing so, this study contributes to the empirical foundation and clinical utility of such programming for the general population.

## Methods

### Participants

A total of 273 adults participated in this study. All participants were recruited from social media (ie, Facebook and Instagram) between May and September 2022, with advertisements targeting a 50% (50/100) female audience. Before enrollment, an online screening questionnaire confirmed eligibility. A total of 529 participants were recruited and screened for this study.

Participants were eligible for participation in this study if they were located in the United States, English-speaking, aged older than 18 years, used an Apple operating system mobile device, and agreed to not alter current mental health treatment, if any. Given the online nature of this study, they were required to verify their identity by uploading a photo of their state-issued identification card. They were also required to complete a decisional balance exercise to ensure a thorough understanding of and willingness to participate in this study. This exercise, based on the work of Goldberg and Kiernan [29], aimed to increase study retention rates by discussing the nature, design, and importance of this study and required potential participants to consider all the pros and cons of participation before deciding whether or not to participate.

Participants were excluded if they had a current or past diagnosis of psychotic disorder or bipolar disorder; if they had started, stopped, or changed doses of psychotropic medication in the past 2 months; or had started or stopped psychotherapy within the past 2 months. They were excluded if they had used the Noom Mood program previously, or were currently using the Noom Weight program.

**Figure 1:**
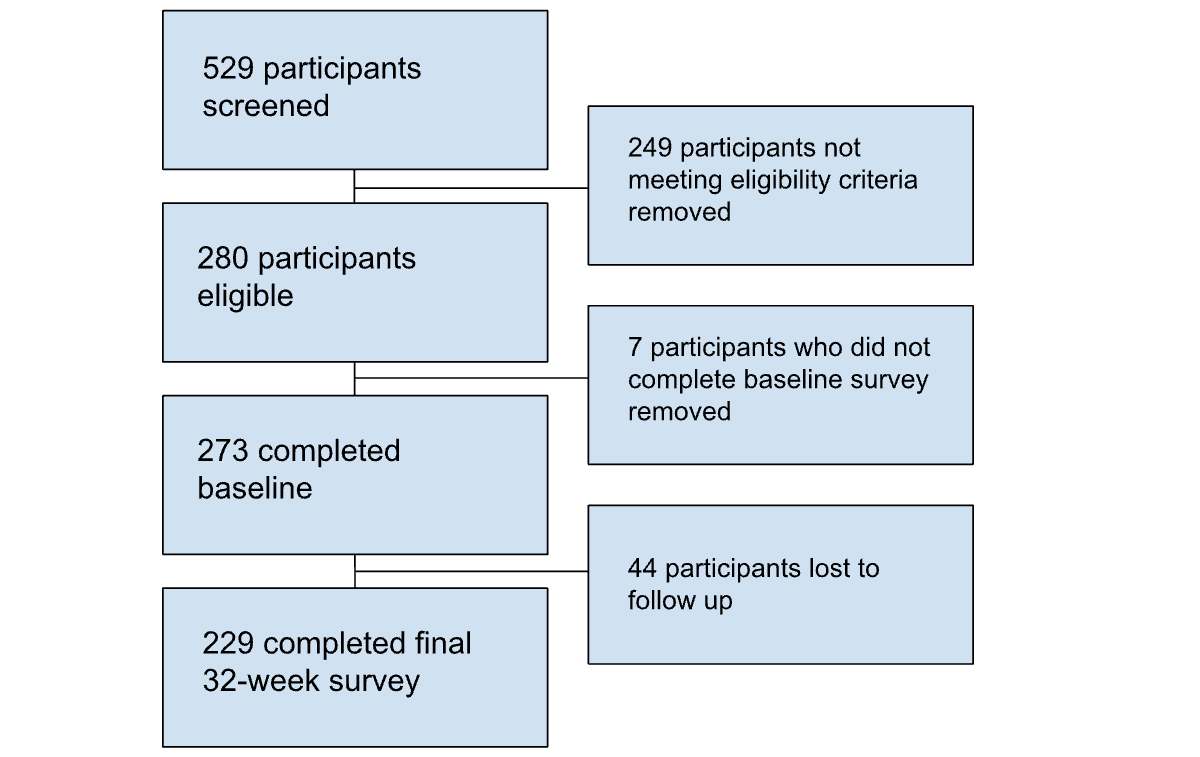
Study flow CONSORT diagram. CONSORT: Consolidated Standards of Reporting Trials.

In total, 351 participants were eligible for participation. Eligible participants were provided informed consent forms and were required to download the app within 14 days to be considered fully enrolled. The final sample included 273 participants who downloaded the program and provided consent (see Figure 1).

### Ethical Considerations

This study was approved by the Advarra Institutional Review Board (Pro00062644).

### Power Analysis and Sample Size Calculation

The sample size was determined based on an estimated medium effect on postintervention scores on our primary outcome, the 7-item generalized anxiety disorder scale (GAD-7), from baseline to end of the program, 95% power, and α set at .05. The effect size is based on an ITT analysis of the GAD-7 (*d*=0.70) from a previous Noom Mood feasibility study [24]. The sample size calculation was conducted using G*Power [30] matched pairs *t* tests (2-tailed). Our recruitment target also accounted for 48% attrition, based on a recent meta-analysis of dropout rates in clinical trials with smartphone apps [31]. Therefore, this study aimed to recruit at least 247 participants in total.

### Recruitment and Eligibility

The eligibility criteria of this study included the ability to understand and provide informed consent, willingness to not alter current mental health treatment (ie, psychotropic medications or psychotherapy), aged at least 18 years, English speaking, located in the United States, iOS user (because certain program features were only available on iPhones at the time of study launch), and successful identity verification. The ineligibility criteria included current or past diagnosis of psychotic disorder or bipolar disorder, past use of the Noom Mood program, starting, changing dose, or stopping psychotropic medication within the past 2 months, starting or stopping psychotherapy within the last 2 months, or current use of the Noom Weight program.

### Procedure

Participants were invited to complete the baseline questionnaire after consenting to participate in this study. As part of this study, participants were required to redeem a unique code and download the Noom Mood app within 14 days of consent, however, there were no further stipulations on participants’ level of engagement with the app. As part of this study, all participants had access to the full Noom Mood program, which consists of 16 weeks of content, at no cost. Participants were surveyed 4, 8, 12, 16, and 32 weeks after baseline. All surveys were administered online via OpenClinica, a HIPAA (Health Insurance Portability and Accountability Act)-compliant electronic data capture platform [32].

### Program

Noom Mood is a commercially available, mobile app–based, mobile mental wellness program that focuses on stress and anxiety management as well as improving overall mental wellness. The program is based on cognitive behavioral therapy, dialectical behavior therapy, acceptance and commitment therapy, and mindfulness-based stress reduction with an emphasis on building a toolkit of coping skills that can be applied to daily life. Participants have access to several program features, including a daily curriculum consisting of psychoeducational articles for users to read, personalized coaching offered through in-app messaging, weekly skills-based activities, and a mood-logging feature. In a previous study of feasibility, acceptability, and preliminary outcomes, significant improvements in anxiety symptoms, perceived stress, depressive feelings, emotion regulation, and optimism were found from baseline to 4-week follow-up. Additionally, learning coping skills (eg, breathing and cognitive reframing techniques), interacting with the program features, and gaining awareness of personal emotions and thought patterns were the features of the program that participants reported benefiting most from [24].

### Measures

#### Overview

The primary outcomes of this study were self-reported measures of symptoms of anxiety, stress, depression, perceived health and quality of life, and mental wellness, detailed below.

##### Anxiety Symptoms (GAD 7)

The GAD-7 [33] assesses the extent to which individuals experience symptoms of anxiety (eg, “feeling nervous, anxious, or on edge”) on a scale of 0 (“not at all”) to 3 (“nearly every day”). Clinical severity was determined using a normed cutoff of 10 for this measure.

##### Depressive Symptoms (8-Item Patient Health Questionnaire Depression Scale)

The 8-item Patient Health Questionnaire depression scale [34] assesses the extent to which participants experience feelings of depression (eg, “feeling down, depressed, or hopeless” or “little interest or pleasure in doing things”) on a scale of 0 (“not at all”) to 3 (“nearly every day”). Clinical severity was determined using a normed cutoff of 10 for this measure.

##### Perceived Stress (10-Item Perceived Stress Scale)

The 10-item Perceived Stress Scale [35] assesses the frequency with which individuals experience various symptoms of stress (eg, “how often have you felt that you were unable to control the important things in your life?”) on a scale of 0 (“never”) to 4 (“very often”).

##### Mental Wellness (14-Item Warwick Edinburgh Mental Well-Being Scale)

The 14-Item Warwick Edinburgh Mental Well-Being scale [36] measures mental wellness. The questions (eg, I have been feeling “optimistic, useful, relaxed”) are answered using a scale of 0 (“none of the time”) to 5 (“all of the time”).

##### Absenteeism, Presenteeism, and Productivity Loss (Sheehan Disability Scale, Modified)

The Sheehan Disability Scale [37] is a scale that measures impairment in functioning (“how many days in the last week did your symptoms cause you to miss school or work or leave you unable to carry out normal daily responsibilities?”). Further, 2 items in particular were used for this study. Absenteeism was measured with the days lost item (“on how many days in the last month did your symptoms cause you to miss school or work or leave you unable to carry out your normal daily responsibilities?”); participants provided a number from 0 to 30. Presenteeism was measured with the days unproductive item (“on how many days in the last month did you feel so impaired by your symptoms, that even though you went to school or work, your productivity was reduced?”) Participants provided a number from 0 to 30. Productivity loss was calculated as the sum of absenteeism and presenteeism, similar to previous work [38,39].

##### Quality of Life (10-Item Patient-Reported Outcomes Measurement Information System Global)

The 10-item Patient-Reported Outcomes Measurement Information System Global scale [40] measures general health care–related quality of life (eg, “how would you rate your physical health?”) on a scale of 1 (“poor”) to 5 (“excellent”).

##### Sleep (Pittsburgh Sleep Quality Index)

The Pittsburgh Sleep Quality Index [41] is a 9-item scale used to measure sleep quality and patterns in adults. The first 4 questions (for instance, “when have you usually gone to bed?”) are free-response questions. The following 4 questions (eg, “during the past month, how often have you taken medicine [prescribed or ‘over the counter’] to help you sleep?”) use a scale of 0 (“not during the past month”) to 3 (“three or more times per week”). The final question (“during the past month, how would you rate your sleep quality overall?”) uses a scale of 0 “very good” to 3 “very bad.”

##### Emotion Regulation (Difficulties in Emotion Regulation Scale)

The Difficulties in Emotion Regulation Scale [42] is an 18-item scale that measures emotional dysregulation (eg, “I pay attention to how I feel”) and uses a scale of 1 “almost never” to 5 “almost always.”

### Statistical Analysis

All analyses were linear mixed-effects models. Dummy codes reflecting comparisons between baseline and all other time points were included as predictors of each primary outcome. Random intercepts were specified for all models. For count data (eg, absenteeism or presenteeism), a Poisson mixed-effects model was used. For categorical outcomes (eg, clinical cutoffs), logistic mixed-effects models were used. Maximum-likelihood estimation was used to account for missing data, which was relatively low (see below).

## Results

### Demographics and Participation

Participant demographic characteristics are presented in Table 1. Of the 273 participants, the mean age was 40.5 (SD 11.7) years. Nearly half (124/273, 45.4%) identified as female, 53.8% (147/273) as male, and 0.7% (2/273) as other, within a predominantly White, non-Hispanic sample (85%). A substantial portion of the sample possessed a college degree (103/273, 37.7%) or graduate degree (81/273, 29.7%). Income distribution spanned a wide range, with the majority reporting annual incomes of US $25,000-$50,000 (44/273, 16.1%), US $50,000-$100,000 (98/273, 35.9%), or US $100,000-$200,000 (70/273, 25.6%). Attrition was minimal and unlikely to introduce bias, with a maximum of 7% missing data reported at all time points.

**Table 1:** Participant demographics (N=273).

Demographics		Value
Age (years), mean (SD)		40.5 (11.8)
**Gender, n (%)**		
	Male	147 (53.8)
	Female	124 (45.4)
	Other	2 (0.7)
**Ethnicity, n (%)**		
	Hispanic	23 (8.4)
	Not Hispanic	245 (90.1)
	Prefer not to say or N/A^a^	5 (1.8)
**Race, n (%)**		
	Asian or Pacific Islander	38 (13.9)
	Black or African American	26 (13.1)
	White	215 (85.3)
	Other	8 (2.9)
	Prefer not to say or N/A	4 (1.5)
**Income, n (%)**		
	Less than US $25,000	23 (8.4)
	US $25,000-$50,000	44 (16.2)
	US $50,000-$100,000	98 (36)
	US $100,000-$200,000	70 (25.7)
	More than US $200,000	22 (8.1)
	Prefer not to say or N/A	16 (5.5)
**Education, n (%)**		
	High school, GED^b^, or less education	15 (5.5)
	Some college or Associate degree	73 (26.7)
	College graduate	103 (37.7)
	Graduate degree	81 (29.7)
	Prefer not to say	1 (0.4)

^a^N/A: not applicable.

^b^GED: General Educational Development.

### Retention

Of the 273 participants who on a baseline survey with retention rates at the 4-week, 8-week, 12-week, 16-week, and 32-week surveys, respectively, were 266/273 (97.4% retention rate from baseline), 255/273 (93.4% retention rate from baseline), 245/273 (89.7% retention rate from baseline), 250/273 (91.6% retention rate from baseline), and 229/273 (83.9% retention rate from baseline).

### Psychological Distress

**Figure 2:**
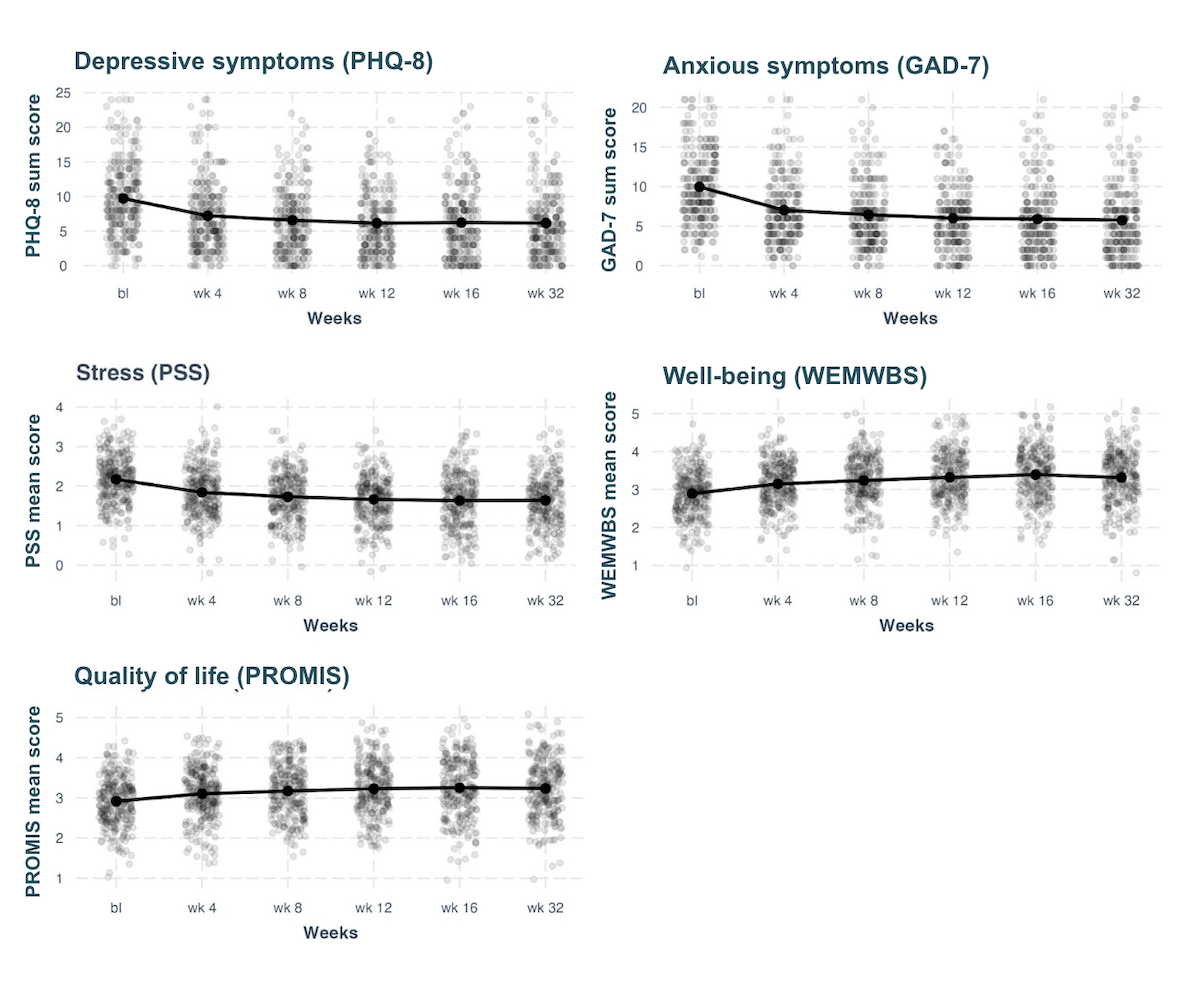
The effects of time on anxiety symptoms, depression symptoms, perceived stress, well-being, and quality of life. Points are jittered to avoid overplotting. bl: baseline; GAD-7: 7-item Generalized Anxiety Disorder Scale; PHQ-8: 8-item Patient Health Questionnaire depression scale; PROMIS: Patient-Reported Outcomes Measurement Information System; PSS: Perceived Stress Scale; WEMWBS: Warwick Edinburgh Mental Well-Being scale.

Statistically significant changes in anxiety symptoms, depression symptoms, perceived stress, well-being, and quality of life were observed by week 4 and maintained through program end (16 weeks) and follow-up (32 weeks, *P*<.001). The largest changes occurred between baseline and 4 weeks, with only small incremental improvements afterward, as shown in Figure 2. Mixed-effects regression models, using random intercepts, were used to assess the impact of time on the aforementioned variables, as depicted in Figure 2 and Tables 2 and 3. All comparisons between program end (16-weeks) and follow-up (32-weeks) were not statistically significant (.29≤ *P* values ≤.99).

**Table 2:** Changes in anxiety symptoms, depression symptoms, perceived stress, well-being, and quality of life at follow-up time points versus baseline (predictors).

		Depressive symptoms				Anxious symptoms				Stress				Well-being				Quality of life		
		Estimates	Standard β	*P* value	Estimates		Standard β	*P* value	Estimates		Standard β	*P* value	Estimates		Standard β	*P* value	Estimates		Standard β	*P* value
Predictors																				
	Intercept	9.72	.55	<.001	9.96		.70	<.001	2.17		.60	<.001	2.89		–.49	<.001	2.92		–.35	<.001
	BL^a^ vs wk 4	–2.51	–.50	<.001	–2.95		–.66	<.001	–0.33		–.51	<.001	0.25		.38	<.001	0.19		.28	<.001
	BL vs wk 8	–3.16	–.63	<.001	–3.52		–.78	<.001	–0.44		–.68	<.001	0.35		.52	<.001	0.25		.38	<.001
	BL vs wk 12	–3.58	–.71	<.001	–3.96		–.88	<.001	–0.5		–.78	<.001	0.43		.64	<.001	0.31		.47	<.001
	BL vs wk 16	–3.50	–.70	<.001	–4.07		–.91	<.001	–0.54		–.84	<.001	0.50		.75	<.001	0.34		.51	<.001
	BL vs wk 32	–3.56	–.71	<.001	–4.22		–.94	<.001	–0.53		–.82	<.001	0.42		.64	<.001	0.32		.48	<.001

^a^BL: baseline.

**Table 3:** Changes in anxiety symptoms, depression symptoms, perceived stress, well-being, and quality of life at follow-up time points versus baseline (random effects).

		Depressive symptoms	Anxious symptoms	Stress	Well-being	Quality of life
**Random effects**						
	σ^2^	8.60	8.53	0.14	0.17	0.11
	^Γ^00	15.56_acce__sscode_	9.55_access__code_	0.24_accesscode_	0.25_accesscode_	0.32_accesscode_
	ICC^a^	0.64	0.53	0.64	0.60	0.74
	N	273_accesscode_	273_accesscode_	272_accesscode_	273_accesscode_	273_accesscode_
Observations		1552	1555	1545	1548	1545
Marginal *R*^2^/conditional *R*^2^		0.065/0.667	0.109/0.579	0.088/0.669	0.062/0.629	0.032/0.743

^a^ICC: intraclass correlation coefficient.

### Clinical Severity

**Figure 3:**
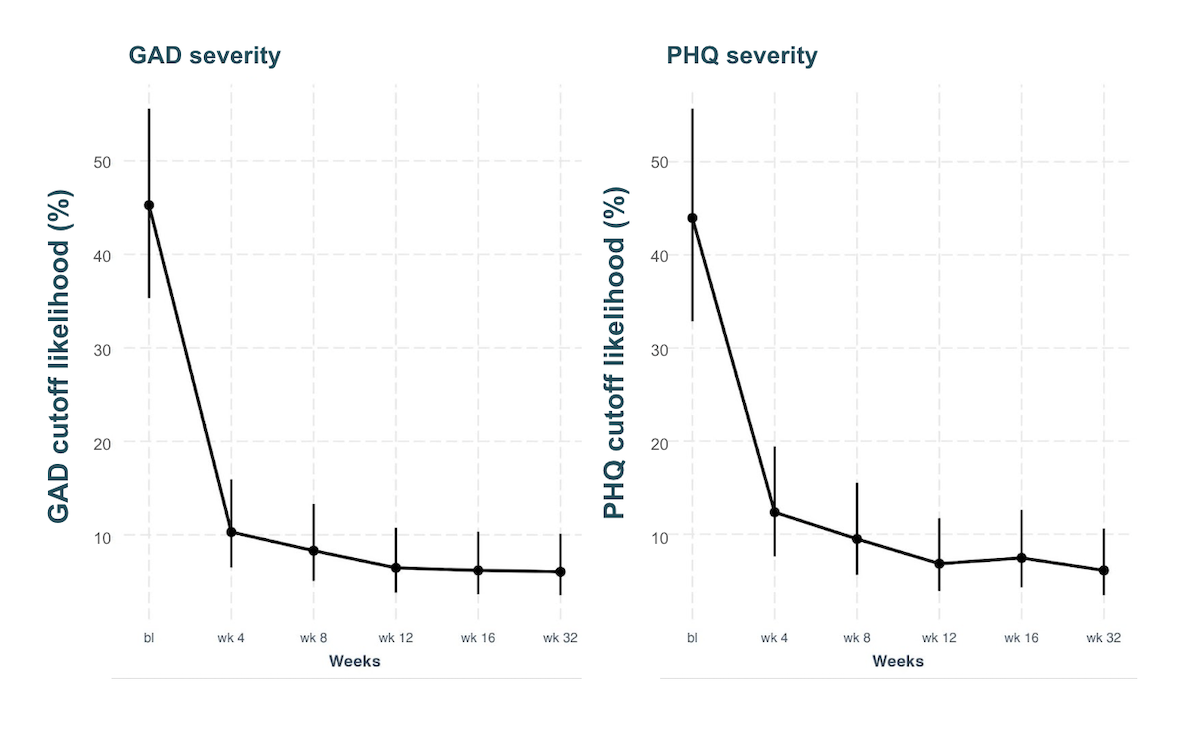
Reductions in the probability of meeting a clinically relevant cutoff score of 10 for GAD and PHQ. Error bars represent SEs. bl: baseline; GAD: Generalized Anxiety Disorder Scale; PHQ: Patient Health Questionnaire depression scale.

At baseline, nearly half of all participants met clinically relevant criteria for anxiety (130/273, 47.6%) and depression (130/273, 47.6%) symptoms. A mixed-effects binomial logistic regression with a random intercept was conducted to analyze changes in clinical severity. Reductions in the probability of meeting clinically relevant criteria for anxiety or depression were significant across all time points (*P*<.001), as shown in Tables 4 and 5. By week 4, the model estimates that only 10% and 12% met the criteria for anxiety or depression, respectively. By week 8, these estimated percentages further decreased to 8% and 9%. The substantial reduction in participants meeting clinical criteria for anxiety or depression was maintained on average through program end (16 weeks) and follow-up (32 weeks), as illustrated in Figure 3.

**Table 4:** Changes in anxiety symptom severity and depression symptom severity at follow-up time points versus baseline (predictors).

		Depressive symptom severity				Anxious symptom severity		
		Odds ratios	Standard β	*P* value	Odds ratios		Standard β	*P* value
Predictors								
	Intercept	0.78	.78	.31	0.83		.83	.37
	BL^a^ vs wk 4	0.18	.18	<.001	0.14		.14	<.001
	BL vs wk 8	0.13	.13	<.001	0.11		.11	<.001
	BL vs wk 12	0.09	.09	<.001	0.08		.08	<.001
	BL vs wk 16	0.10	.10	<.001	0.08		.08	<.001
	BL vs wk 32	0.08	.08	<.001	0.08		.08	<.001

^a^BL: baseline.

**Table 5:** Changes in anxiety symptom severity and depression symptom severity at follow-up time points versus baseline (random effects).

		Depressive symptom severity		Anxious symptom severity
**Random effects**				
	σ^2^		3.29	3.29
	^Γ^00		6.35_accesscode_	_4.45accesscode_
	ICC^a^		0.66	0.58
	N		273_accesscode_	273_accesscode_
Observations		1552		1555
Marginal *R*^2^/conditional *R*^2^		0.072/0.683		0.098/0.617

^a^ICC: intraclass correlation coefficient.

### Overall Well-Being

**Figure 4:**
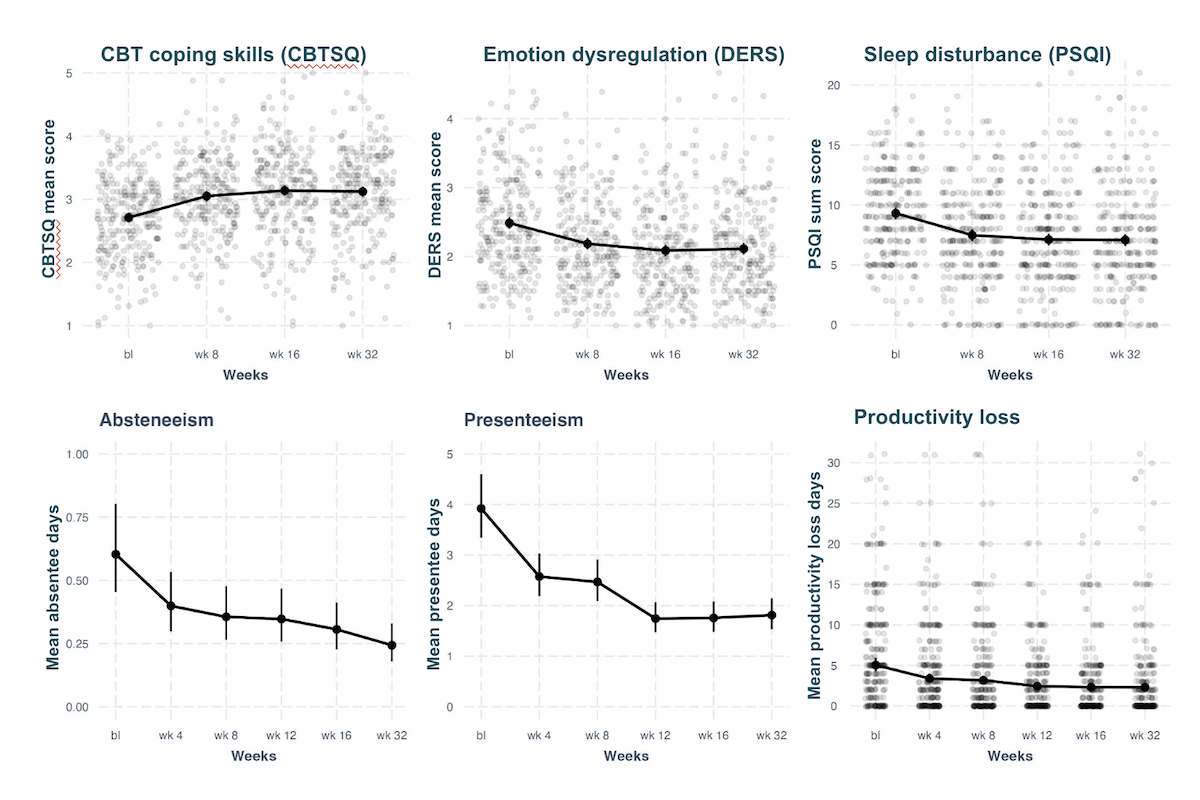
Plots of mixed-effects models for changes to well-being over time. bl: baseline; CBT: cognitive behavioral therapy; CBTSQ: Cognitive Behavioral Therapy Skills Questionnaire; DERS: Difficulties in Emotion Regulation Scale; PSQI: Pittsburgh Sleep Quality Index.

Secondary effects of the intervention on coping, emotion regulation, sleep, and workplace absenteeism or presenteeism were examined via separate models for each variable. Absenteeism and presenteeism days were aggregated to form a comprehensive productivity loss outcome. A 0-inflated Poisson mixed-effects model with a random intercept was used for absenteeism and presenteeism. By program end (16 weeks), participants gained 2.57 productive work days from baseline, with this number remaining relatively stable (2.23 days) at follow-up (32 weeks). Effects across all well-being variables exhibited positive and significant trends at all time points (*P*<.001), as summarized in Tables 6 and 7 and displayed in Figure 4.

**Table 6:** Changes to well-being at follow-up time points versus baseline (predictors).

		Coping			Emotion dysregulation			Sleep disturbance			Absenteeism			Presenteeism			Productivity loss		
		Estimates	Standard β	*P* value	Estimates	Standard β	*P* value	Estimates	Standard β	*P* value	Incidence rate ratios	Standard β	*P* value	Incidence rate ratios	Standard β	*P* value	Incidence rate ratios	Standard β	*P* value
**Predictors**																			
	Intercept	2.71	–.42	<.001	2.49	.38	<.001	931	.37	<.001	0.60	.60	.001	3.92	3.92	<.001	5.04	5.04	<.001
	BL^a^ vs wk 16										0.66	.66	<.001	0.66	.66	<.001	0.67	.67	<.001
	BL vs wk 4	0.34	.51	<.001	–0.30	–.44	<.001	–1.83	–.44	<.001	0.59	.59	<.001	0.63	.63	<.001	0.63	.63	<.001
	BL vs wk 32										0.58	.58	<.001	0.44	.44	<.001	0.48	.48	<.001
	BL vs wk 8	0.43	.63	<.001	–0.40	–.58	<.001	–2.20	–.52	<.001	0.51	.51	<.001	0.45	.45	<.001	0.46	.46	<.001
	BL vs wk 12	0.41	.61	<.001	–0.37	–.54	<.001	–2.23	–.53	<.001	0.40	.40	<.001	0.46	.46	<.001	0.46	.46	<.001

^a^BL: baseline.

**Table 7:** Changes to well-being at follow-up time points versus baseline (random effects).

			Coping		Emotion dysregulation		Sleep disturbance		Absenteeism	Presenteeism		Productivity loss
**Random effects**												
	σ^2^	0.19		0.17		7.15		1.28		0.35	0.27	
	^Γ^00	0.24_accesscode_		0.28_accesscode_		9.62_accesscode_		3.64_accesscode_		1.53_accesscode_	1.65_accesscode_	
	ICC^a^	0.56		0.63		0.57		0.74		0.82	0.86	
	N	273_accesscode_		273_accesscode_		273_accesscode_		273_accesscode_		273_accesscode_	273_accesscode_	
Observations			1030		1030		1100		1478	1479		1476
Marginal *R*^2^/conditional *R*^2^			0.068/0.593		0.055/0.649		0.048/0.594		0.016/0.743	0.044/0.824		0.040/0.863

^a^ICC: intraclass correlation coefficient.

## Discussion

### Principal Findings

This study contributes to research on mobile mental wellness interventions by examining the efficacy and postintervention outcomes of the Noom Mood program for the general population. The investigation addresses gaps in the existing literature by examining the impact of this type of intervention on mental health and well-being outcomes both postintervention and after a 16-week maintenance period. Using a prospective 1-arm design, our study evaluated the effects of Noom Mood on an array of mental health indicators and well-being measures, spanning from baseline assessment to a follow-up assessment 32 weeks later (16 weeks after the conclusion of the intervention phase). Promising results were seen in terms of improvements in mental health, workplace productivity, and well-being outcomes.

Our study benefited from several notable strengths. First, a major strength of this study was the achievement of retention rates ranging from 85% to 98%. Low retention rates are a widely acknowledged barrier to understanding and applying the results of digital health research. Other digital studies have often found the most successful retention rates (48%-56%) to still remain suboptimal [31,43]. The higher retention rates observed within this study are likely related to the screening practices used, such as the decisional balance exercise, which are designed to both select for and enhance participant motivation and commitment at this study’s start [29]. An additional strength of this study is the generalizability of the results. Given that we did not select individuals with a certain score on mental health measures, the results support the intervention’s ability to reduce distress and enhance well-being for this quite large group of individuals. Furthermore, we did not use a certain engagement threshold for this study, meaning that participants were able to use the app as much or as little as they liked, and the results remained promising. Finally, this type of mobile app–based intervention has a large potential for scalability.

### Mental Health

Mobile mental wellness apps have shown promising potential in providing support and resources for individuals experiencing stress, symptoms of depression, and symptoms of anxiety [21,44,45]. Further, a study by Economides et al [25] reported substantial and sustained improvements in depression and anxiety symptoms 6 months after an 8-week therapist-led smartphone-based intervention for elevated symptoms of anxiety and depression. Likewise, a recent systematic review and meta-analysis of mobile mental wellness interventions suggested the high potential of these interventions to improve (or significantly support the improvement of) mental health challenges such as depression and anxiety [28]. These findings underscore the potential for a prolonged positive impact of mental wellness interventions on major mental health dimensions.

Similarly, a recent systematic review and meta-analysis of 9 randomized controlled trials (RCTs) including 1837 participants also found small to medium effect sizes of mobile mental wellness apps on anxious symptoms, though when compared with an active control only small effect sizes were found [21]. Similarly, a meta-analysis of 18 RCTs including 3414 participants found that depressive symptoms were reduced significantly following a digital mental health intervention when compared with a control condition; effect sizes were small, however, when compared to active control conditions [45].

Our findings showed statistically significant reductions within 4 weeks of program initiation for both anxiety and depression symptoms, assessed using the GAD-7 and 8-item Patient Health Questionnaire depression scales respectively. These initial improvements are maintained throughout the program, from the intervention’s conclusion at 16 weeks to the 32-week follow-up assessment. Outside of symptom reduction, we observed a significant reduction in the proportion of participants meeting clinically relevant anxiety and depression symptom criteria at all assessment points. Our findings align with other published results, however, the outcomes of the meta-analyses highlight the need for a study comparing Noom Mood with an active control condition.

### Workplace Productivity and Accessibility

Previous studies have shown a clear connection between mental health and workplace productivity [13,46-49]. Further, a cross-sectional study found the mean days of absenteeism and presenteeism were significantly higher among participants with moderate or high psychological distress compared to low distress [47], and a recent multi-arm, pilot RCT examining the outcome of digital interventions among working adults showed promising preliminary findings indicating improved depressive symptoms, well-being, and functioning following a digital intervention [48].

In parallel, this study showed the potential of a mental wellness intervention to reduce both symptoms of depression and decreased workplace absences along with improved workplace focus. The personalized nature and convenience of mobile interventions allow them to be positioned as promising tools for reaching individuals with limited access to conventional mental health services.

Furthermore, to rationalize employer investment in mobile mental wellness programs, it is necessary to demonstrate their effectiveness in not only improving employee health and well-being but also in saving money. Previous studies have indicated that digital mental wellness interventions can reduce weekly costs by US $155.82 over 8 weeks [49]. Given the anticipated costs to employers associated with anxiety, stress, and depressive symptoms, there is a clear indication that scalable digital interventions such as Noom Mood have the potential to demonstrate significant cost savings within the workplace.

### Well-Being

Several studies have cited the scarcity of research looking at various dimensions of well-being within the context of self-guided mobile mental wellness interventions [27,28]. The literature that does exist is somewhat ambivalent, with some studies finding improvements in well-being as a result of a digital mental wellness intervention and some not. For instance, a recent systematic review and meta-analysis found that digital mental wellness apps, when compared to controls, showed small effects for reducing mental health symptoms and improving well-being, but a medium effect for emotion regulation [28]. Another recent RCT found that the group using a mindfulness app for 4 weeks saw small reductions in stress and depression, but students who practiced more did not experience additional improvement in well-being [50].

Our study reveals enhancements in well-being dimensions including coping skills, emotion regulation, and sleep quality, and suggests that mobile mental wellness programs are not only beneficial in terms of symptom reduction but also support a variety of well-being dimensions. However, given the existing literature, and the limitations of this study’s design, more rigorous testing is needed to better understand this association.

### Limitations

Limitations of this study include the absence of a control group, lack of sample diversity, reliance on self-report measures, potential selection bias from recruitment via social media ads, and the potential impact of high retention rates on outcomes’ representativeness. Importantly, results should be interpreted with caution as this study’s design did not use a control group, meaning results could in part be due to the passage of time alone. This study advances understanding of the effects of a commercially available mental wellness program on various mental health outcomes and contributes potential insights into the program’s utility for the general population; however, robust research is needed to affirm the program’s efficacy, particularly across diverse populations.

### Conclusions

In conclusion, this single-arm prospective study examined 16-week and 32-week outcomes of the Noom Mood self-guided smartphone-based mental wellness program, including a 16-week maintenance period. Our outcomes showed positive impacts on mental health and well-being dimensions, both in terms of symptom reduction and broader psychosocial aspects, though outcomes are limited in scope due to the lack of a control arm in this study’s design. The results highlight significant improvements across all mental health and well-being outcomes at program end (16 weeks), which extend to postprogram follow-up (32 weeks). Significant improvements emerge as early as the first follow-up assessment, conducted 4 weeks after program initiation. These findings contribute to the evidence base of mental wellness programs and address gaps in the literature concerning maintenance and the comprehensive effects of a digital self-guided mental wellness intervention.

Future studies must include an RCT study design and a study population spanning diverse groups, accounting for race and ethnicity, education level, and income level. Additionally, future research should aim to understand the specific mechanisms underlying the observed improvements as well as explore the program’s scalability and feasibility within real-world settings. Further research investigating the program’s potential for long-term adherence and sustained outcomes may additionally provide valuable insights for both practitioners and researchers. Lastly, a direct comparison of the effectiveness of the Noom Mood program with similar interventions may shed light on the relative advantages and disadvantages of different approaches in the field of mobile mental wellness.

## Abbreviations

GAD-7: 7-item Generalized Anxiety Disorder Scale
HIPAA: Health Insurance Portability and Accountability Act
RCT: randomized controlled trial
